# Comparison of traditional anesthesia method and jet injector anesthesia method (MadaJet XL®) for Nexplanon® insertion and removal

**DOI:** 10.1186/s40834-020-00104-x

**Published:** 2020-02-24

**Authors:** G. Anthony Wilson, Julie W. Jeter, William S. Dabbs, Amy Barger Stevens, Robert E. Heidel, Shaunta’ M. Chamberlin

**Affiliations:** Department of Family Medicine, University of Tennessee Graduate School of Medicine, 1924 Alcoa Highway, Box U-67, Knoxville, TN 37920 USA

**Keywords:** Local anesthetic, Nexplanon®, Patient anxiety

## Abstract

**Background:**

This study compared a needle-free anesthesia method with traditional local anesthesia for insertion and removal of Nexplanon® long-acting removable contraceptive device. In our clinic, patients often avoid this highly effective form of contraception due to fear of needles. We sought to determine if patients perceived a difference in pain with the injection, anxiety level or pain with the procedure when local anesthesia was given with a needle v/s a needle-free jet injector device.

**Methods:**

Patients were randomly assigned to one of two groups: jet injector or needle lidocaine delivery. Outcomes were ease of use, patient anxiety level, painfulness, and efficacy of anesthesia method.

**Results:**

Patient pain perception with administration of jet injector lidocaine was statistically lower than traditional needle with no difference in anxiety or ease of use, or efficacy of the anesthesia.

**Conclusion:**

The jet injector device is a reasonable alternative to needle injection delivery of anesthesia prior to insertion/removal of Nexplanon® device. Further studies may determine whether this needle-free alternative for administration of local anesthetic would result in more women choosing Nexplanon® as a contraceptive method.

## Background

As with many procedures [1, 2], patients often cite a fear of needles as a major reason to decline Nexplanon® placement. Nexplanon® is a long-acting removable contraceptive device that is traditionally inserted in the upper arm under local anesthesia using a needle to inject a lidocaine solution [3]. A jet injector device that injects lidocaine under high pressure without the use of needles has been studied in other medical settings, including dental [1] and urologic [2] procedures, as well as other procedures requiring local anesthetic [4]. The objective of this study was to determine if the jet injection method of local anesthesia is effective for removal and insertion of the Nexplanon® device, whether the pain of the injection of lidocaine differed between the methods of delivery, and whether the presence or absence of needles in the anesthesia method affected patient anxiety level.

## Methods

All adult women of childbearing age who were undergoing insertion or removal of a Nexplanon® device for contraception at a residency-based Family Medicine clinic were invited to be a part of the study. Any persons who declined to be a part of the study had the device inserted by established protocol with needle anesthesia and their data were not used. Expedited IRB approval was obtained prior to starting the study. Patients were randomized via random computerized assignment to one of two methods of anesthesia: The intervention group received 1% lidocaine delivered to the site of insertion or removal via Jet-injector device; the control group received 1% lidocaine using a needle injection. For our study, a spring-loaded jet injector was used. Gas/air powered jet injectors are also available. Per protocol for the respective anesthetic devices, 1–2 ml was used with needle-injected anesthesia, and 8 to 10 jets of lidocaine (each 0.1 ml) were used along the insertion or removal tract for jet injected anesthesia. No differentiation in anesthetic dose was made between insertion and removal as similar amounts of anesthetic are routinely used for both procedures. A patient survey (Table 1) was administered by the investigator 5 to 10 min after the procedure was finished. The provider performing the procedure also answered questions (Table 1) related to the perceived patient experience and ease of use of the respective delivery method.

**Table 1:** Patient and Provider Questionnaire

Questions for patients	
PQ1. Before your procedure, were you worried that the procedure might be painful?	
1-Not at all worried	
2-Slightly worried	
3-Moderately worried	
4-Very worried	
5-Extremely worried	
PQ2. When you saw the needle (or jet injector device), did you become anxious?	
1-Not at all anxious at all	
2-Slightly anxious	
3-Moderately anxious	
4-Very anxious	
5-Extremely anxious	
PQ 3. Did you experience pain with the numbing injection?	
1-No pain at all	
2-Slight pain	
3-Moderate pain	
4-Very painful	
5-Extremely painful	
PQ4. Did you experience pain when the Nexplanon was inserted or removed?	
1-No pain at all	
2-Slight pain	
3-Moderate pain	
4-Very painful	
5-Extremely painful	
Questions for doctors	
DQ1. Was the anesthesia method easy to use?	
1-Very difficult to use	
2-Difficult to use	
3-Fairly easy to use	
4-Easy to use	
5-Very easy to use	
DQ2. Did the patient experience discomfort with the lidocaine injection?	
1-No discomfort at all	
2-Slight discomfort	
3-Moderate discomfort	
4-Serious discomfort	
5-Extreme discomfort	
DQ3. Did the method of anesthesia provide adequate anesthesia for the placement or removal of the Nexplanon®?	
1-Very poor	
2-Poor	
3-Fair	
4-Good	
5-Excellent	

## Statistical methods

The distributions of survey questions were assessed for the statistical assumption of normality using skewness and kurtosis statistics (Table 2). Levene’s Test of Equality of Variances was used to check for the statistical assumption of homogeneity of variance. Between-subject statistics were used to compare the needle injection group versus the jet injector group on the survey questions. When statistical assumptions were met, parametric independent samples *t*-tests were used to compare the groups on the continuous survey item responses. When either or both statistical assumptions were violated, non-parametric Mann-Whitney *U* tests were used for between-subject comparisons. Statistical significance was assumed a Bonferroi-adjusted alpha value of 0.007 to account for increased experiment-wise error rates when testing multiple hypotheses concurrently. All analyses were conducted using SPSS Version 25 (Armonk, NY: IBM Corp.). The study was adequately powered.

**Table 2:** Descriptive Statistics for Between-Subjects Comparisons of Treatment Groups

Survey Item	Needle Injection (*n* = 17)	Jet Injector (*n* = 22)	*p*-value
PQ 1	2.00 (3.00)**	2.00 (1.25)**	0.488
PQ 2	2.18 (0.95)*	1.50 (0.67)*	0.013
PQ 3	1.88 (0.70)*	1.32 (0.48)*	0.005***
PQ 4	1.00 (1.00)**	1.00 (0.00)**	0.058
DQ 1	5.00 (1.00)**	5.00 (0.00)**	0.116
DQ 2	2.00 (0.61)*	1.36 (0.49)*	0.001***
DQ 3	5.00 (1.00)**	5.00 (0.00)**	0.006***

Note: * *M* (SD), ** Median (IQR), *** *p* < 0.007

## Results

Thirty-nine patients were enrolled, 17 randomized to the lidocaine injection with needle and 22 to the lidocaine jet injector. Means + SD and medians with interquartile ranges, in addition to *p*-values can be found in Table 2. Patients seemed to have the same level of concern prior to the procedure, with no statistical or numerical difference in patient question (PQ) 1. A Significant difference was found between the treatment groups for PQ 3, suggesting patients in the jet injector group were less likely to experience pain with the numbing procedure. Although no significant difference was seen between groups for PQ 2 or 4, a potential Type II error was detected. Providers felt that each method of lidocaine delivery was equally convenient (doctor question (DQ) 1). They also perceived the patients in the jet injector group experienced less pain with the administration of anesthesia compared to the needle group (DQ2). Additionally, a significant difference was found between groups for adequacy of anesthesia for the procedure with the jet injector group more consistently being rated as no pain (DQ3).

## Discussion

No prior study has compared local anesthesia delivered with needle versus jet injector for pain with injection, anxiety with the anesthesia method, or efficacy of anesthetic, specifically in regards to insertion or removal of Nexplanon®. In this study, patients had significantly less pain when the local anesthetic was delivered via jet injector. Both traditional needle and jet injector delivery methods produced the desired effect of adequate anesthesia with no significant difference. Additionally, providers did not perceive a difference in ease of use for either method. One limitation of this study is that neither the doctors nor the patients were blinded to the anesthesia method, which could lead to a confirmation bias. Additionally, patients enrolled had already made the decision to utilize Nexplanon® as their choice of contraception, which may have made them less concerned about the anesthesia method or the insertion procedure itself. Future studies are needed and could seek to compare responses to existing validated instruments for pain and situational anxiety. The jet injector device incurs an initial cost ($662.00 in our experience) and early training in use, with minimal ongoing cost, namely expenses in sterilization of the device after use.

## Conclusions

While Nexplanon® is highly efficacious, easily accessible, and immediate acting [5], future studies ascertaining whether a patient’s fear of needles may cause them to reject this reliable means of contraception would be helpful. Offering an alternative, less painful method of anesthesia has the potential to increase acceptance of point-of-care insertion of this highly effective contraceptive device.

## Data Availability

The datasets used and/or analyzed during the current study are available from the corresponding author on reasonable request.
